# Supplementary material for: Revolutionizing colorectal cancer detection: A breakthrough in microbiome data analysis
Source: PLoS One. 2025 Jan 29;20(1):e0316493. doi: 10.1371/journal.pone.0316493 (PMC11778789; doi:10.1371/journal.pone.0316493)
Supplement: Attachment — Submitted filename: maniscript_plos.pdf [file pone.0316493.s001.pdf]

Revolutionizing Colorectal Cancer Detection: A Breakthrough in Microbiome Data Analysis

This is the review report for the manuscript Revolutionizing Colorectal Cancer Detection: A Breakthrough in Microbiome Data Analysis submitted to PLOS ONE. The paper highlights the transformative impact of Next Generation Sequencing (NGS) on clinical diagnosis and personalized medicine through high-throughput microbiome data. Overcoming challenges in traditional statistical and machine learning methods, the study introduces an innovative feature engineering technique that combines two sets of features, resulting in a new dataset. This approach markedly enhances the performance of Deep Neural Network (DNN) algorithms in detecting colorectal cancer using gut microbiome data, showcasing a substantial contribution to addressing the complexities of microbiome data analysis and bolstering the efficacy of deep learning methods in disease detection. There are several drawbacks of the proposed procedure. Here are my comments to justify its usefulness of the proposed procedure:

1. Authors needs to improved introduction to discuss the usefulness of the approach and some contemporary work.
2. The authors needs to highlight the usefulness of the approach in analyzing the high/large-dimensional microbiome data analysis.
3. The paper exhibits subpar writing, characterized by the recurrence of an unsubstantiated idea lacking credible evidence.
4. The author should explicitly articulate the rationale for employing chained normalization, rank transformation, feature extension, and feature selection within the specified application context.
5. Crucially, the justification for employing a deep neural network is warranted in the context of a study with a large sample size. However, the author has utilized a dataset featuring a sample size of only 512 with 324 features, which is insufficient for training a deep neural network. Author should talk about the generalization of the approach.
6. In the analysis of microbiome data, normalization is applied to overcome the constraint of not observing absolute abundance data. The author should explicitly demonstrate the rationale behind employing various normalization techniques. Currently, using two different normalization methods seems unwarranted, particularly in the context of microbiome data analysis.
7. To better facilitate the manuscript review and reproducibility of the analysis, authors should share the analysis script over any open-source platform.
8. The authors are attempting to compare a modified version of their own methodology. Given that this is not the inaugural model capable of performing the classification task, they ought to incorporate other methods in the model comparison to illustrate the novelty of their methodology.
