# Supplementary material for: Revolutionizing colorectal cancer detection: A breakthrough in microbiome data analysis
Source: PLoS One. 2025 Jan 29;20(1):e0316493. doi: 10.1371/journal.pone.0316493 (PMC11778789; doi:10.1371/journal.pone.0316493)
Supplement: Attachment — Submitted filename: Response to Reviewers.docx [file pone.0316493.s002.docx]

**Response to Reviewers’ Comments**

**(**Manuscript. Ref. No.: **PONE-D-23-19630)**

Title: **Revolutionizing colorectal cancer detection: a breakthrough in microbiome data analysis**

Dear Editor:

We appreciate the time and effort exerted by the editor and referees in reviewing this manuscript. We are very thankful to the reviewers for their deep and insightful review. Our manuscript was revised to reflect their useful suggestions and comments. We hope that the paper will be considered worthy of publication as all of the comments have been accommodated. Our responses to their specific comments, suggestions and queries are presented below.

**Review # 1 Comments**

**Comment 1.1**: Clarification of Research Objectives: It is important to further clarify the research objectives at the beginning of the paper. Make sure to elaborate on why reducing dimensions and processing microbiome data is crucial in the context of colorectal cancer (CRC). Explain in more detail why improvements in CRC classification are needed.

**Response1.1**: We appreciate the reviewer's insightful comments and recognize the importance of clarifying the research objectives at the outset of the paper. In response, we have elaborated on the significance of reducing dimensions and processing microbiome data within the framework of colorectal cancer (CRC) detection (line 83 to 86).

**Comment 1.2**: Explanation of Method Selection Process: The authors can provide a better understanding of why the hybrid feature processing technique was chosen for use in this research. Are there strong theoretical reasons or previous studies that support its use?

**Response1.2**: We appreciate the opportunity to address your concerns regarding the method selection process, particularly regarding the rationale behind the adoption of the hybrid feature processing technique. The reason for using a hybrid method is to combine the strengths of individual methods in order to enhance model’s performance. While our method is novel, we have cited appropriate literature to back up our claim as shown in line 67 to 70

**Comment 1.3**: Clarification of Hybrid Feature Processing Technique: The paper includes the use of hybrid feature processing techniques. However, it would be better if the authors provide more in-depth details on how these techniques are specifically implemented. This includes what parameters are used (if any), how these parameters are chosen, and whether there are specific steps taken to optimize these techniques.

**Response1.3**: We appreciate the reviewer's insightful comments on the need to clarifications on the Hybrid Feature Processing Technique. We have expanded our text in line 229 to 231 that previous studies were used as the basis for identifying the optimum configuration of the proposed method. The previous study used a brute force method to identify optimum setups. A brute force method is necessitated by the fact it is currently not possible to predict the performance of a normalisation or of combinations thereof due the un predictable nature of underlying data distributions. Therefor the aim this work is demonstrate that using various permutations of chained normalisation methods to create additional features which are then subjected to feature selection, can significtly improve the predictive performance of model (also, line 324 to 327)

**Comment 1.4**: Deeper Interpretation: To improve this paper, a deeper interpretation of the results is needed. The authors should explain why there is a difference in performance between the various normalization methods. Are there any specific factors that influence these results? Furthermore, is there an explainable relationship between data distribution attributes such as kurtosis, skewness, and AUC?

**Response1.4:** Thank you for your valuable feedback. In response to the suggestion, we have revised the manuscript from line 454 to 457, 493 to 394, 585 to 587

**Comment 2.1**: Authors needs to improved introduction to discuss the usefulness of the approach and some contemporary work.

**Response 2.1:**	Thank you for your valuable feedback. In response to your suggestion, we have revised the introduction to better highlight the usefulness of our approach and discuss contemporary work. Specifically, we have included additional details that underscore the challenges DL methods face when applied to microbiome data, particularly in dealing with high dimensionality and noise, which often degrade model performance. We also reference recent studies that have attempted to mitigate these issues using feature extraction techniques like autoencoders and unsupervised binning, as well as feature selection methods like filter-based approaches. Despite these advancements, we highlight that many of these existing methods address the challenges in a piecemeal manner, often failing to provide comprehensive solutions for dimensionality reduction and feature relevance. We also draw attention to baseline work proposing sequential normalization methods, which, while a step forward, do not fully resolve the issues at hand. Our study aims to build on these foundations by offering a more integrated approach to address both dimensionality and noise, contributing to the robustness of DL models in disease detection (Line 53 to 63).

**Comment 2.2**: The authors need to highlight the usefulness of the approach in analysing the high/large-dimensional micro-biome data analysis.

**Response 2.2:** Thank you for your valuable feedback regarding the need to emphasize the usefulness of our approach in analysing high-dimensional microbiome data. We appreciate the opportunity to clarify this aspect in our manuscript. We have updated the manuscript in line 76 to 77, 83 to 86, 150 to 152, 342 to 343, 590 to 591 to indicate that by using feature selection our method addressing the issue of high data dimensionality

**Comment 2.3:** The paper exhibits subpar writing, characterized by the recurrence of an unsubstantiated idea lacking credible evidence.

**Response 2.3:** Thank you for your constructive feedback. We acknowledge the concern regarding the recurrence of an unsubstantiated idea in the manuscript. We have revised the relevant sections to provide stronger, credible evidence by inserting appropriate citations and clearer justification for the ideas presented. In doing so, we have ensured that all claims are well-supported by appropriate references and data.

**Comment 2.4**: The author should explicitly articulate the rationale for employing chained normalization, rank transformation, feature extension, and feature selection within the specified application context.

**Response 2.4:** Thank you for your thoughtful feedback. We recognize the need to clarify the rationale behind employing chained normalization, rank transformation, feature extension, and feature selection. In the revised manuscript, we have provided a detailed explanation for each of these techniques in the context of our application starting from line 585 to 591.

- Chained normalization was employed to standardize the data across different scales, ensuring consistency and improving model convergence.
- Rank transformation was introduced to reduce the impact of outliers and to focus on the relative importance of features.
- Feature extension aimed to enrich the feature space, allowing the model to capture complex relationships between variables.
- Feature selection was crucial in reducing dimensionality, minimizing noise, and retaining the most relevant features, given the high-dimensional nature of the dataset.

These techniques were chosen to optimize model performance and enhance generalization in the specified context. We hope the additional explanation strengthens the manuscript."

**Comment 2.5**: Crucially, the justification for employing a deep neural network is warranted in the context of a study with a large sample size. However, the author has utilized a dataset featuring a sample size of only 512 with 324 features, which is insufficient for training a deep neural network. Author should talk about the generalization

of the approach.

**Response 2.5:** Thank you for highlighting the concern regarding the dataset size and the justification for employing a deep neural network. We acknowledge that deep neural networks typically perform better with larger sample sizes. However, in our study, we applied specific techniques to address this limitation, such as regularization, and careful tuning of the model's complexity (Line 332 to 337). These strategies were employed to prevent overfitting and improve the generalization of the model, despite the relatively small sample size.

**Comment 2.6**: In the analysis of microbiome data, normalization is applied to overcome the constraint of not observing absolute abundance data. The author should explicitly demonstrate the rationale behind employing various normalization techniques. Currently, using two different normalization methods seems unwarranted, particularly in the context of microbiome data analysis.

**Response 2.6:** Thank you for your valuable feedback. We would like to clarify the rationale behind employing multiple normalization methods in our analysis. As outlined in the manuscript, we utilized a sequential arrangement of normalization techniques, where the output of one method is fed into the next. This approach was designed to address the complex characteristics of microbiome data, such as its high dimensionality and variability in distribution.

Research has demonstrated that no single normalization method consistently outperforms others across all datasets due to the diverse nature of underlying data distributions (as cited). Likewise, there is no universally optimal combination of methods for use in a chained sequence. Thus, our choice to apply multiple normalization techniques was motivated by the need to capture different aspects of the data that a single method might overlook. We have revised the manuscript to further clarify this reasoning and ensure that the rationale for using sequential normalization techniques is more explicitly justified. We appreciate your thorough review and hope that this explanation resolves your concerns.

**Comment 2.7**. To better facilitate the manuscript review and reproducibility of the analysis, authors should share the analysis script over any open-source platform.

**Response 2.7:** Thank you for your suggestion regarding the sharing of the analysis script to enhance review facilitation and reproducibility. In response, we have uploaded the analysis script to GitHub, an open-source and included the link in the revised manuscript (<https://github.com/diskava/Revolutionizing-colorectal-cancer-detection-A-breakthrough-in-microbiome-data-analysis.git> ). The link has also been inserted in the manuscript in line The script contains all relevant details of the data preprocessing, model training, and evaluation steps to ensure transparency and reproducibility. We hope this will assist in a more thorough review of the manuscript and ease the reproducibility of our results.

**Comment 3.1**: Performance of the DNN model requires estimation of TP, TN, FP, and FN for obtaining TPR, TNR, and FPR. Require clear explanation of the DNN model based on these parameters.

**Response 3.1:** Thank you for your valuable feedback. To respond to the comment, the confusion matrices for methods in dataset 1 have been added and analysed (line 517 to 538).

**Comment 3.2**: Missing details of the gut microbial composition in CRC and healthy conditions. **Response 3.2:**	We appreciate the reviewer’s insightful suggestion regarding the inclusion of biomarkers for colorectal cancer detection. However, the primary focus of the present study is on improving deep neural network performance for classification using gut microbiome data, specifically through feature engineering and normalization methods. As such, we did not explore the identification or validation of specific biomarkers in this work. We agree that investigating microbial abundance as biomarkers for colorectal cancer is an important future direction, but it is beyond the current scope of this study. We hope this explanation is acceptable and appreciate your understanding. Also, we have updated the conclusion to address these concerns (line 614 to 619)

**Comment 3.3:** Chain normalization was used, which includes MMN, PSN, VSS, and ZSN methods. MMADN method could improve the model's accuracy.

**Response 3.3:** We appreciate the valuable feedback. As such have added text to show how MMADN was excluded from the manuscript. The facts are that in our preliminary work MMADN did not show promising results. That is why it was exclude as shown in line 217 to 221

**Comment 3.4 :** Additional analysis required for experimental validation of the DL-based colorectal cancer detection.

**Response 3.4:** We are grateful for this remark. We wish to acknowledge that Additional analysis is important for model validation. However, we wish to point that the paper uses an additional dataset besides the dataset proposed in our work. See line 179 to 180 and line 540 to 565

**Comment 3.5:** Here it was mentioned about working with a default learning rate, please add the exact value. Can also compare the training accuracy with changing this learning rate.

**Response 3.5:** We are grateful for this remark. We wish to acknowledge that there was a lapse in our earlier statement which states that the default learning rate was used when in the actual sense, the model used is based and indeed the learning rate was adopted from the experiment in the cited baseline work. As such, the text has been modified to show that that the learning rate and other parameters of the model has been adopted from previously published work. The modifications to the manuscript are in line 332 to 338

**Comment 3.6:** A more detailed explanation of the feature extraction stage in the DNN model would contribute significantly to the understanding of the model architecture. Could the authors elaborate on the key aspects and methodologies employed during this stage?

**Response 3.6:** We appreciate the valuable feedback. While the proposed method does not directly use feature extraction, we assume the reviewer meant feature selection. As such we have outlined how L2 regularization was used in feature selection in our work ( Line 324 to 327)

**Comment 3.7:** While the authors have implemented noise reduction techniques, it is pertinent to discuss the potential drawbacks of such methods. Could the authors address concerns regarding the inadvertent elimination of essential data elements during the noise reduction process and elaborate on how these concerns were mitigated in their study?

**Response 3.6:** We appreciate the reviewer's insightful comment regarding the potential drawbacks of noise reduction techniques, particularly the inadvertent elimination of essential data elements. In our study, we were aware of this risk and took deliberate steps to mitigate it as explained in Line 592 to 600. By reversing the order of these steps in each execution path, we generated two distinct feature sets, which helped retain vital information from the original dataset. This redundancy through transformation further ensured that key data points were preserved even as noise was minimized.

Additionally, feature selection was applied to the consolidated dataset post-transformation to address high dimensionality, prioritizing features that contributed most to model performance. This selection process helped safeguard against the loss of important data elements by emphasizing those features that were most relevant to the classification task.

While we acknowledge the potential for the inadvertent removal of significant data during noise reduction, the combination of multiple transformation paths and feature selection effectively mitigated this risk in our study. We believe that these steps not only reduced noise but also preserved essential elements critical to the classification of colorectal cancer using gut microbiome data. Experimental results have demonstrated that these strategies improved model performance, further supporting the efficacy of the approach.

**Comment 3.8:** Add one figure showing the detailed DNN model for better understanding of the architecture.

**Response 3.8:** Thank you for your valuable feedback regarding the inclusion of a figure to illustrate the DNN model. We agree that a visual representation will enhance the reader's understanding of the architecture. In response, we have created a detailed figure of the DNN model, which we have included in the manuscript as Figure 2

**Comment 3.9:** May conclude with some biomarkers (Microbial abundance) for colorectal cancer detection.

**Response 3.9:** We appreciate the reviewer’s insightful suggestion regarding the inclusion of biomarkers for colorectal cancer detection. However, the primary focus of the present study is on improving deep neural network performance for classification using gut microbiome data, specifically through feature engineering and normalization methods. As such, we did not explore the identification or validation of specific biomarkers in this work. We agree that investigating microbial abundance as biomarkers for colorectal cancer is an important future direction, but it is beyond the current scope of this study. We hope this explanation is acceptable and appreciate your understanding. Also, we have updated the conclusion to include this concern

**Comment 3.10:** The manuscript has several typo errors, ("Raw and pre-Ranking" and "Raw and Post-Ranking"); pre is written in lowercase, whereas the Post is in uppercase. Please maintain a consistency

**Response 3.10:** Thank you for your insightful feedback. We have corrected the inconsistencies by capitalizing each word and using nouns instead of the present continuous form. "Pre-Rank" and "Post-Rank" are now consistently formatted throughout the manuscript.
